# Awareness of Palestinians about lung cancer symptoms: a national cross-sectional study

**DOI:** 10.1186/s12890-022-01923-1

**Published:** 2022-04-08

**Authors:** Mohamedraed Elshami, Hanan Abukmail, Wafa Aqel, Mohammed Alser, Ibrahim Al-Slaibi, Hanan Shurrab, Shahd Qassem, Faten Darwish Usrof, Malik Alruzayqat, Roba Nairoukh, Ahmad Mansour, Rahaf Kittaneh, Nawras Sawafta, Yousef M. N. Habes, Obaida Ghanim, Wesam Almajd Aabed, Ola Omar, Motaz Daraghmeh, Jomana Aljbour, Razan Elian, Areen Zuhour, Haneen Habes, Mohammed Al-Dadah, Nasser Abu-El-Noor, Bettina Bottcher

**Affiliations:** 1 Division of Surgical Oncology, Department of Surgery, University Hospitals Cleveland Medical Center, 11100 Euclid Avenue, Lakeside 7100, Cleveland, OH 44106 USA; 2 Ministry of Health, Gaza, Palestine; 3 Faculty of Medicine, Islamic University of Gaza, Gaza, Palestine; 4 Faculty of Medicine, Al-Quds University, Jerusalem, Palestine; 5 The United Nations Relief and Works Agency for Palestine Refugees in the Near East, Gaza Strip, Palestine; 6 Almakassed Hospital, Jerusalem, Palestine; 7 Faculty of Pharmacy, Al-Azhar University of Gaza, Gaza, Palestine; 8 Department of a Medical Laboratory Sciences, Faculty of Health Sciences, Islamic University of Gaza, Gaza City, Palestine; 9 Faculty of Dentistry, Al-Quds University, Jerusalem, Palestine; 10 Faculty of Nursing, An Najah National University, Nablus, Palestine; 11 Faculty of Dentistry, Al Azhar University of Gaza, Gaza, Palestine; 12 Faculty of Medicine, Al Najah National University, Nablus, Palestine; 13 Faculty of Nursing, Islamic University of Gaza, Gaza, Palestine

**Keywords:** Lung cancer, Awareness, Symptoms, Survival, Early presentation, Palestine, Educational interventions, Health education

## Abstract

**Background:**

The majority of lung cancer (LC) cases are diagnosed at an advanced stage. Poor awareness of LC symptoms is a contributor to late diagnosis. This study aimed to assess the awareness of LC symptoms among Palestinians, and to examine the factors associated with displaying good awareness.

**Methods:**

Participants were recruited from hospitals, primary healthcare centers and public spaces using convenience sampling. A translated-into-Arabic version of the validated LC awareness measure was used to assess recognition of 14 LC symptoms. One point was given for each recognized symptom. The total score was calculated and categorized based on the number of symptoms recognized: poor (0–4), fair (5–9), and good (10–14). Multivariable logistic regression was used to examine the association between participant characteristics and having good awareness. The multivariable analysis adjusted for age-group, gender, education, monthly income, occupation, residence, marital status, any chronic disease, knowing someone with cancer, smoking history, and site of data collection.

**Results:**

Of 5174 potential participants approached, 4817 completed the questionnaire (response rate = 93.1%) and 4762 were included in the final analysis. Of these, 2742 (56.9%) were from the West Bank and Jerusalem (WBJ) and 2020 (43.1%) were from the Gaza Strip. Participants from the WBJ were older, had higher monthly income but lower education, and suffered from more chronic diseases. The most recognized respiratory LC symptom was ‘worsening in an existing cough’(n = 3884, 81.6%) while the least recognized was ‘a cough that does not go away for two or three weeks’(n = 2951, 62.0%). The most recognized non-respiratory LC symptom was ‘persistent tiredness or lack of energy’(n = 3205, 67.3%) while the least recognized was ‘persistent shoulder pain’(n = 1170, 24.6%).

A total of 2466 participants (51.8%) displayed good awareness of LC symptoms. Participants from both the Gaza Strip and the WBJ had similar likelihoods to have good awareness levels. Factors associated with a higher likelihood to display good awareness included female gender, having post-secondary education, being employed, knowing someone with cancer, and visiting hospitals and primary healthcare centers.

**Conclusion:**

About half of the study participants displayed a good level of awareness of LC symptoms. Further improvement in public awareness of LC symptoms by educational interventions might reduce LC mortality by promoting early diagnosis.

**Supplementary Information:**

The online version contains supplementary material available at 10.1186/s12890-022-01923-1.

## Introduction

Lung cancer (LC) is the leading cause of cancer-related deaths worldwide and the second most commonly diagnosed cancer [1, 2]. In 2020, over 1.7 million deaths and 2.2 million new LC cases were estimated [1, 2]. The overall cancer-related mortality is potentially higher in low- and middle-income countries [3, 4]. In Palestine, a lower-middle-income country, LC is also the leading cause of cancer-related deaths (17.3%) and the second commonly diagnosed cancer (11.4%) [5]. Palestine has an age-standardized mortality rate of 19.2 per 100,000 persons, which is higher than that of some other countries in the region, such as Egypt (7.2 per 100,000 persons) [6], Iraq (11.1 per 100,000 persons) [7], Jordan (14.3 per 100,000 persons) [8], and Lebanon (16.6 per 100,000 persons) [9].

LC is one of the main causes of avoidable mortality around the world [10]. Notably, more than 50% of LC patients are diagnosed at an advanced stage which lowers their chances for survival [10]. Poor awareness of LC symptoms is considered one of the contributing factors to late presentation [11]. Early diagnosis plays a crucial role in cancer control especially in low- and middle-income countries [3]. In countries with low-resource settings (e.g., Palestine), fragile health systems, economic difficulties, and poor health literacy are factors that impede achieving optimal cancer care services [12]. Cancer outcomes could be improved by raising the recognition of symptoms, which in turn may facilitate early seeking to medical advice [13].

There are different potential symptoms associated with LC including cough, shortness of breath, chest pain, coughing up blood, and finger clubbing [14]. Promoting the awareness of LC symptoms may enhance early presentation and diagnosis leading to better survival outcomes [15–17]. Identifying the population’s level of knowledge about LC symptoms could help in designing educational interventions to promote public awareness. This eventually may lead to lower morbidity and mortality rates associated with LC particularly in countries with low-resource settings like Palestine.

A previous study found that 47.7% of Palestinians in the West Bank were smokers and 74.4% of the those started smoking when they aged 18 years or younger [18]. Men had higher smoking rates than women. The most common methods of smoking among Palestinians were cigarettes and waterpipes [18], which are considered significant risk factors for LC [19–21].

Prior studies from Palestine investigated the awareness of different cancers including breast, colorectal, cervical, and ovarian cancers [22–27]. However, to the best of our knowledge, awareness of LC has not been studied. Given the high prevalence of smoking in Palestine [18] and the lack of baseline information about LC awareness, this national study aimed to (i) evaluate the awareness of Palestinians about LC symptoms, (ii) examine if there is a difference in the awareness level between the two main areas in Palestine: the Gaza Strip vs. the West Bank and Jerusalem (WBJ), and (iii) identify the sociodemographic factors associated with good level of awareness of LC symptoms.

## Materials and methods

### Study design and population

This was a national cross-sectional study. Data were collected between July 2019 and March 2020 in the two main areas of Palestine: the WBJ and the Gaza Strip. Adult Palestinians (≥ 18 years) were the target population. Recruitment took place at governmental hospitals, primary healthcare centers and public spaces, such as markets, malls, restaurants, parks, mosques, churches, city centers, transportation stations and others. Participants were excluded if they had a nationality other than Palestinian, working or studying in a health-related field, and visiting oncology departments or clinics at the time of data collection.

### Sampling methods

A convenience sampling technique was used to recruit eligible participants from governmental hospitals, primary healthcare centers, and public spaces in 11 (out of 16) governorates across Palestine. These governorates have a population of 4,644,074, which makes up about 90% of the total population of Palestine [28]. The inclusion of participants from different places was intended to increase the diversity of the study cohort to resemble the Palestinian community [22–24].

### Questionnaire and data collection

A modified version of the LC Awareness Measure (LCAM) was used to collect data from the designated sites. The LCAM is a validated instrument for assessing public awareness of LC [11]. The questionnaire went through the process of translation and adaptation of instruments as recommended by the World Health Organization (WHO) [29]. Two bilingual healthcare professionals translated the original LCAM into Arabic, which was subsequently back-translated into English by another two bilingual healthcare professionals. Three experts in the fields of thoracic oncology, public health, and survey design evaluated the Arabic version of the LCAM for content validity and accuracy of translation. Following that, a pilot study (n = 68) was conducted to assess the clarity of questions in the Arabic LCAM. The final analysis did not include the questionnaires of the pilot study. The internal consistency of the Arabic LCAM was assessed using Cronbach’s Alpha, which reached a good value of 0.846.

The Arabic LCAM included two sections. The first section described the sociodemographic characteristics of the study participants. The second section evaluated the level of awareness of participants about the 14 LC symptoms using a 5-point Likert scale (1 = strongly disagree, 5 = strongly agree). All of the 14 symptoms were used in the original LCAM [30]. However, the questions in the original LCAM with yes/no/unknown responses were converted into 5-point Likert scale questions to reduce the potential of participants answering questions at random. This was followed by converting the participants’ responses to correct/incorrect responses similar to previous studies [22–25, 31]. The questionnaire can be accessed, please see Additional file 1.

Potential participants were approached by data collectors in the waiting areas at hospitals and primary healthcare centers as well as in public spaces, such as parks, restaurants, malls, transportation stations, and others. Eligible individuals were invited to participate in the study by completing the questionnaire in a face-to-face interview. The data collectors had a medical background and received a special training to learn how to recruit participants, conduct the interviews, and facilitate the completion of the Arabic LCAM. Data were collected utilizing the secure, user-friendly data collection tool ‘Kobo Toolbox’ that was accessed via smartphones [32].

### Statistical analysis

The likelihood to develop LC increases markedly starting from the age of 45 [33]. Therefore, the continuous variable of age was categorized into two categories using this cutoff: 18–44 years and ≥ 45 years. The minimum wage in Palestine is 1450 NIS, about $450 [34]. The continuous variable of monthly income was categorized into two categories using the minimum wage as the cutoff: < 1450 NIS and ≥ 1450 NIS.

The median [interquartile range [IQR] was used to describe continuous, non-normally distributed characteristics of the study participants and Kruskal–Wallis test was used to perform baseline comparisons. Frequencies and percentages were used to describe categorical characteristics and Pearson's Chi-square test was used to perform baseline comparisons.

The recognition of each LC symptom was evaluated using a question based on a 5-point Likert scale (1 = strongly disagree, 5 = strongly agree). Answering with ‘strongly agree’ or ‘agree’ was considered as a correct response while answering with ‘strongly disagree’, ‘disagree’, or ‘not sure’ was considered as an incorrect response. For each correctly recognized LC symptom, one point was given. LC symptoms were further categorized into two categories: (i) respiratory and (ii) non-respiratory symptoms. Frequencies and percentages were used to describe the recognition of LC symptoms and Pearson's Chi-Square test was used to make comparisons between participants from the Gaza Strip vs. the WBJ. After that, bivariable and multivariable logistic regression analyses were run to examine the association between recognizing each LC symptom and participant characteristics. The factors included in the multivariable analysis were age group, gender, educational level, monthly income, occupation, place of residency, marital status, having a chronic disease, knowing someone with cancer, smoking history, and site of data collection. This model was determined a priori based on previous studies [11, 12, 35–37].

In consistence with previous studies [22–27, 31], a scoring system was utilized to assess the participants’ awareness level of LC symptoms. For each correctly recognized LC symptom, one point was given. The total score (ranging from 0 to 14) was calculated and categorized based on the number of symptoms recognized correctly into three categories: poor (0 to 4), fair (5 to 9), and good (10 to 14). The awareness level of LC symptoms displayed by the participants from the Gaza Strip vs. the WBJ was compared using Pearson's Chi-Square test. This was followed by running bivariable and multivariable logistic regression analyses to examine the association between having good awareness of LC symptoms and participant characteristics. Please see Additional file 2 for the results of bivariable analyses.

Missing data were handled using a complete case analysis approach, where they occurred completely at random. Data were analyzed using Stata software version 16.0 (StataCorp, College Station, Texas, United States).

## Results

### Participant characteristics

A total of 4817 participants, out of 5174 approached, completed the questionnaire (response rate = 93.1%). The final analysis included 4762 questionnaires (24 did not meet inclusion criteria and 31 had missing data): 2742 participants were from the WBJ and 2020 were from the Gaza Strip. The median age [IQR] for all participants was 32.0 years [24.0, 44.0] (Table 1). Participants from the WBJ were older, had higher monthly income but lower levels of education, smoked cigarettes or shisha more frequently, and suffered from more chronic diseases than participants from the Gaza Strip.

**Table 1:** Characteristics of study participants

Characteristic	Total (n = 4762)	Gaza Strip (n = 2020)	WBJ (n = 2742)	*p* value
Age, median [IQR]	32.0 [24.0, 44.0]	30.0 [24.0, 40.0]	34.0 [24.0, 47.0]	< 0.001
Age group, n (%)				
18 to 44	3572 (75.0)	1634 (80.9)	1938 (70.7)	< 0.001
45 or older	1190 (25.0)	386 (19.1)	804 (29.3)	
Female gender, n (%)	2618 (55.0)	1086 (53.8)	1532 (55.9)	0.15
Educational level, n (%)				
Secondary or below	2375 (49.9)	955 (47.3)	1420 (51.8)	0.002
Post-secondary	2387 (50.1)	1065 (52.7)	1322 (48.2)	
Occupation, n (%)				
Unemployed/housewife	2003 (42.1)	970 (48.0)	1033 (37.7)	< 0.001
Employed	2160 (45.4)	814 (40.3)	1346 (49.1)	
Retired	111 (2.3)	46 (2.3)	65 (2.4)	
Student	488 (10.2)	190 (9.4)	298 (10.8)	
Monthly income ≥ 1450 NIS, n (%)	3241 (68.1)	683 (33.8)	2558 (93.3)	< 0.001
Marital status, n (%)				
Single	1480 (31.1)	641 (31.7)	839 (30.6)	0.07
Married	3117 (65.5)	1323 (65.5)	1794 (65.4)	
Divorced/Widowed	165 (3.5)	56 (2.8)	109 (4.0)	
Having a chronic disease, n (%)	1032 (21.7)	313 (15.5)	719 (26.2)	< 0.001
Knowing someone with cancer, n (%)	2571 (54.0)	1045 (51.7)	1526 (55.7)	0.007
Ever smoked, n (%)				
Cigarettes	1127 (23.7)	417 (20.6)	710 (25.9)	< 0.001
Waterpipe (Shisha)	499 (10.5)	142 (7.0)	357 (13.0)	< 0.001
Site of data collection, n (%)				
Public Spaces	1920 (40.3)	784 (38.8)	1136 (41.4)	< 0.001
Hospitals	1628 (34.2)	651 (32.2)	977 (35.7)	
Primary healthcare centers	1214 (25.5)	585 (29.0)	629 (22.9)	

n, number of participants; IQR, interquartile range; WBJ, West Bank and Jerusalem

### Recognition of LC symptoms

The most recognized respiratory LC symptom was ‘worsening or change in an existing cough’ (n = 3884, 81.6%) followed by ‘coughing up blood’ (n = 3776, 79.3%) (Table 2). The least recognized respiratory LC symptom was ‘a cough that does not go away for two or three weeks’ (n = 2951, 62.0%). The most recognized non-respiratory LC symptom was ‘persistent tiredness or lack of energy’ (n = 3205, 67.3%). The least recognized non-respiratory LC symptom was ‘persistent shoulder pain’ (n = 1170, 24.6%).

**Table 2:** Recognition of lung cancer symptoms

Symptom	Total (n = 4762) n (%)	Gaza Strip (n = 2020) n (%)	WBJ (n = 2742) n (%)	*p* value
Respiratory symptoms				
Worsening or change in an existing cough	3884 (81.6%)	1614 (80.0%)	2270 (82.8%)	0.011
Coughing up blood	3776 (79.3%)	1536 (76.0%)	2240 (81.7%)	< 0.001
Persistent shortness of breath	3528 (74.1%)	1483 (73.4%)	2045 (74.6%)	0.36
An ache or pain when breathing	3487 (73.2%)	1470 (72.8%)	2017 (73.6%)	0.54
Persistent chest pain	3440 (72.2%)	1466 (72.6%)	1974 (72.0%)	0.66
Painful cough	3356 (70.5%)	1373 (68.0%)	1983 (72.3%)	0.001
Persistent (3 weeks or longer) chest infection	3265 (68.6%)	1374 (68.0%)	1891 (69.0%)	0.49
A cough that does not go away for two or three weeks	2951 (62.0%)	1193 (59.1%)	1758 (64.1%)	< 0.001
Non-respiratory symptoms				
Persistent tiredness or lack of energy	3205 (67.3%)	1400 (69.3%)	1805 (65.8%)	0.011
Developing an unexplained loud, high-pitched sound when breathing	3040 (63.8%)	1261 (62.4%)	1779 (64.9%)	0.082
Loss of appetite	3021 (63.4%)	1311 (64.9%)	1710 (62.4%)	0.072
Unexplained weight loss	2697 (56.6%)	1114 (55.1%)	1583 (57.7%)	0.075
Changes in the shape of fingers or nails	1657 (34.8%)	768 (38.0%)	889 (32.4%)	< 0.001
Persistent shoulder pain	1170 (24.6%)	539 (26.7%)	631 (23.0%)	0.004

n, number of participants; WBJ, West Bank and Jerusalem

### Good awareness and its associated factors

A total of 2466 participants (51.8%) displayed good awareness (i.e., prompt recognition of more than nine out of 14 LC symptoms) (Table 3). No difference was found between the proportion of participants demonstrating good awareness in the Gaza Strip and WBJ (50.6% vs. 52.6%). On the multivariable analysis, female gender, having post-secondary education, being employed, knowing someone with cancer, and visiting hospitals and primary healthcare centers were all associated with an increase in the likelihood of having a good level of awareness of LC symptoms (Table 4).

**Table 3:** awareness level of lung cancer symptoms among study participants

Level	Total n (%)	Gaza Strip n (%)	WBJ n (%)	p-value
Poor (0–4 symptoms)	516 (13.0%)	279 (13.9%)	336 (12.3%)	0.21
Fair (5–9 symptoms)	1,681 (35.3%)	718 (35.5%)	963 (35.1%)	
Good (10–14 symptoms)	2,466 (51.7%)	1023 (50.6%)	1443 (52.6%)	

n, number of participants, WBJ, West Bank and Jerusalem

**Table 4:** Bivariable and multivariable logistic regression analyzing factors associated with having a good awareness of lung cancer symptoms

Characteristic	Good awareness			
	COR (95% CI)	P	AOR (95% CI)*	P
Age group				
18 to 44	Ref	Ref	Ref	Ref
45 or older	1.01 (0.88–1.15)	0.91	1.06 (0.90–1.24)	0.52
Gender				
Male	Ref	Ref	Ref	Ref
Female	1.27 (1.14–1.43)	< 0.001	1.30 (1.10–1.53)	0.002
Educational level				
Secondary or below	Ref	Ref	Ref	Ref
Post-secondary	1.45 (1.29–1.62)	< 0.001	1.49 (1.31–1.70)	< 0.001
Occupation				
Unemployed/housewife	Ref	Ref	Ref	Ref
Employed	1.01 (0.89–1.14)	0.88	1.22 (1.04–1.43)	0.015
Retired	1.15 (0.78–1.69)	0.48	1.25 (0.82–1.90)	0.30
Student	1.05 (0.86–1.28)	0.64	1.17 (0.92–1.48)	0.21
Monthly income				
< 1450 NIS	Ref	Ref	Ref	Ref
≥ 1450 NIS	1.18 (1.04–1.33)	0.010	1.18 (1.00–1.40)	0.052
Marital status				
Single	Ref	Ref	Ref	Ref
Married	1.05 (0.93–1.19)	0.44	0.94 (0.81–1.10)	0.44
Divorced/Widowed	0.97 (0.71–1.34)	0.86	0.96 (0.67–1.36)	0.80
Residency				
Gaza Strip	Ref	Ref	Ref	Ref
WBJ	1.08 (.97–1.22)	0.18	0.99 (0.85–1.16)	0.92
Having a chronic disease				
No	Ref	Ref	Ref	Ref
Yes	1.01 (.88–1.16)	0.91	1.0 (.85–1.18)	0.98
Knowing someone with cancer				
No	Ref	Ref	Ref	Ref
Yes	1.28 (1.14–1.43)	< 0.001	1.41 (1.25–1.59)	< 0.001
Ever smoked cigarettes and/or shisha				
No	Ref	Ref	Ref	Ref
Yes	0.84 (0.74–0.95)	0.005	0.94 (0.80–1.09)	0.40
Site of data collection				
Public Spaces	Ref	Ref	Ref	Ref
Hospitals	1.58 (1.38–1.80)	< 0.001	1.80 (1.57–2.07)	< 0.001
Primary healthcare centers	1.90 (1.64–2.20)	< 0.001	2.20 (1.87–2.59)	< 0.001

COR = crude odds ratio, AOR = adjusted odds ratio, CI = confidence interval, WBJ = West Bank and Jerusalem

*Adjusted for age-group, gender, educational level, occupation, monthly income, marital status, residency, having a chronic disease, knowing someone with cancer, smoking history, and site of data collection

### Association between recognizing respiratory LC symptoms and participant characteristics

Participants with post-secondary education were more likely than participants with lower education to recognize all respiratory LC symptoms (Table 5). In addition, participants recruited from hospitals or primary healthcare centers were more likely than participants recruited from public spaces to recognize all respiratory LC symptoms. Participants who knew someone with cancer were more likely than participants who did not to recognize all respiratory LC symptoms except ‘coughing up blood’ for which no associated difference was found. There was no associated difference in the likelihood to recognize all respiratory LC symptoms between smokers and non-smokers.

**Table 5:** Multivariable logistic regression analyzing factors associated with the recognition of respiratory symptoms of lung cancer

Characteristic	Worsening or change in an existing cough		Coughing up blood		Persistent shortness of breath		An ache or pain when breathing	
	AOR (95% CI)*	p-value	AOR (95% CI)*	p-value	AOR (95% CI)*	p-value	AOR (95% CI)*	p-value
Age group								
18 to 44	Ref	Ref	Ref	Ref	Ref	Ref	Ref	Ref
45 or older	0.81 (0.66–0.99)	0.041	0.87 (0.71–1.06)	0.16	0.99 (0.82–1.19)	0.89	0.86 (0.72–1.03)	0.09
Gender								
Male	Ref	Ref	Ref	Ref	Ref	Ref	Ref	Ref
Female	1.29 (1.04–1.61)	0.021	1.12 (0.91–1.37)	0.30	0.92 (0.76–1.12)	0.42	1.16 (0.96–1.40)	0.12
Educational level								
Secondary or below	Ref	Ref	Ref	Ref	Ref	Ref	Ref	Ref
Above secondary	1.79 (1.51–2.11)	< 0.001	1.50 (1.29–1.76)	< 0.001	1.71 (1.47–1.98)	< 0.001	1.40 (1.21–1.61)	< 0.001
Occupation								
Unemployed/housewife	Ref	Ref	Ref	Ref	Ref	Ref	Ref	Ref
Employed	1.11 (0.90–1.36)	0.35	1.28 (1.05–1.56)	0.013	1.12 (0.93–1.34)	0.24	1.18 (0.99–1.41)	0.07
Retired	0.71 (0.44–1.15)	0.17	1.12 (0.68–1.84)	0.66	1.22 (0.74–1.99)	0.44	1.42 (0.89–2.29)	0.15
Student	1.25 (0.90–1.75)	0.19	1.33 (0.99–1.80)	0.06	1.57 (1.17–2.09)	0.003	1.32 (0.99–1.76)	0.05
Monthly income								
< 1450 NIS	Ref	Ref	Ref	Ref	Ref	Ref	Ref	Ref
≥ 1450 NIS	1.26 (1.01–1.56)	0.037	1.21 (0.99–1.48)	0.06	1.25 (1.03–1.51)	0.024	1.12 (0.93–1.35)	0.24
Marital status								
Single	Ref	Ref	Ref	Ref	Ref	Ref	Ref	Ref
Married	0.85 (0.70–1.04)	0.10	1.06 (0.88–1.27)	0.57	0.99 (0.831.17)	0.88	0.80 (0.68–0.96)	0.014
Divorced/Widowed	0.87 (0.56–1.36)	0.55	1.66 (1.04–2.64)	0.033	0.84 (0.57–1.22)	0.36	0.83 (0.57–1.23)	0.36
Residency								
Gaza Strip	Ref	Ref	Ref	Ref	Ref	Ref	Ref	Ref
WBJ	1.09 (0.89–1.33)	0.41	1.27 (1.05–1.54)	0.015	0.98 (0.82–1.17)	0.80	1.02 (0.85–1.22)	0.83
Having a chronic disease								
No	Ref	Ref	Ref	Ref	Ref	Ref	Ref	Ref
Yes	1.27 (1.03–1.57)	0.026	1.10 (0.90–1.34)	0.36	0.92 (0.77–1.11)	0.39	0.87 (0.73–1.04)	0.13
Knowing someone with cancer								
No	Ref	Ref	Ref	Ref	Ref	Ref	Ref	Ref
Yes	1.26 (1.08–1.47)	0.003	1.07 (0.92–1.23)	0.38	1.40 (1.22–1.60)	< 0.001	1.43 (1.25–1.64)	< 0.001
Ever smoked cigarettes and/or shisha								
No	Ref	Ref	Ref	Ref	Ref	Ref	Ref	Ref
Yes	0.93 (0.77–1.14)	0.50	0.97 (0.80–1.18)	0.79	0.90 (0.75–1.07)	0.23	0.96 (0.80–1.14)	0.64
Site of data collection								
Public Spaces	Ref	Ref	Ref	Ref	Ref	Ref	Ref	Ref
Hospitals	1.45 (1.22–1.73)	< 0.001	1.36 (1.15–1.61)	< 0.001	1.69 (1.45–1.99)	< 0.001	1.73 (1.48–2.02)	< 0.001
Primary healthcare centers	1.69 (1.37–2.09)	< 0.001	1.56 (1.28–1.90)	< 0.001	2.03 (1.68–2.44)	< 0.001	2.10 (1.75–2.53)	< 0.001

Characteristic	Persistent chest pain		Painful cough		Persistent (3 weeks or longer) chest infection		A cough that does not go away for two or three weeks	
	AOR (95% CI)*	p-value	AOR (95% CI)*	p-value	AOR (95% CI)*	p-value	AOR (95% CI)*	p-value
Age group								
18 to 44	Ref	Ref	Ref	Ref	Ref	Ref	Ref	Ref
45 or older	1.12 (0.93–1.34)	0.23	0.96 (0.81–1.15)	0.67	1.07 (0.90–1.27)	0.48	1.09 (0.92–1.29)	0.31
Gender								
Male	Ref	Ref	Ref	Ref	Ref	Ref	Ref	Ref
Female	0.96 (0.79–1.15)	0.63	1.12 (0.93–1.34)	0.24	1.08 (0.90–1.30)	0.4	1.19 (1.00–1.41)	0.051
Educational level								
Secondary or below	Ref	Ref	Ref	Ref	Ref	Ref	Ref	Ref
Above secondary	1.27 (1.10–1.47)	0.001	1.49 (1.29–1.71)	< 0.001	1.54 (1.34–1.76)	< 0.001	1.48 (1.29–1.69)	< 0.001
Occupation								
Unemployed/housewife	Ref	Ref	Ref	Ref	Ref	Ref	Ref	Ref
Employed	1.12 (0.94–1.34)	0.21	1.10 (0.93–1.31)	0.28	1.17 (0.99–1.39)	0.07	1.25 (1.06–1.47)	0.007
Retired	1.22 (0.76–1.98)	0.41	0.98 (0.63–1.54)	0.94	1.11 (0.71–1.73)	0.66	1.13 (0.74–1.73)	0.58
Student	1.42 (1.08–1.87)	0.021	1.36 (1.03–1.79)	0.029	1.21 (0.93–1.58)	0.15	1.32 (1.03–1.69)	0.031
Monthly income								
< 1450 NIS	Ref	Ref	Ref	Ref	Ref	Ref	Ref	Ref
≥ 1450 NIS	1.20 (0.99–1.44)	0.06	1.22 (1.02–1.46)	0.034	1.20 (1.00–1.43)	0.051	1.18 (1.00–1.40)	0.06
Marital status								
Single	Ref	Ref	Ref	Ref	Ref	Ref	Ref	Ref
Married	0.86 (0.73–1.02)	0.08	0.82 (0.70–0.97)	0.023	0.88 (0.75–1.04)	0.13	0.85 (0.72–0.99)	0.036
Divorced/Widowed	0.87 (0.59–1.27)	0.46	0.83 (0.57–1.22)	0.35	0.64 (0.44–0.92)	0.015	0.84 (0.59–1.20)	0.34
Residency								
Gaza Strip	Ref	Ref	Ref	Ref	Ref	Ref	Ref	Ref
WBJ	0.88 (0.74–1.05)	0.15	1.13 (0.95–1.34)	0.17	0.97 (0.82–1.15)	0.70	1.13 (0.96–1.33)	0.14
Having a chronic disease								
No	Ref	Ref	Ref	Ref	Ref	Ref	Ref	Ref
Yes	0.96 (0.80–1.15)	0.66	1.01 (0.85–1.21)	0.92	0.93 (0.78–1.11)	0.43	0.99 (0.84–1.17)	0.90
Knowing someone with cancer								
No	Ref	Ref	Ref	Ref	Ref	Ref	Ref	Ref
Yes	1.42 (1.24–1.62)	< 0.001	1.36 (1.20–1.55)	< 0.001	1.22 (1.07–1.38)	0.003	1.16 (1.03–1.31)	0.017
Ever smoked cigarettes and/or shisha								
No	Ref	Ref	Ref	Ref	Ref	Ref	Ref	Ref
Yes	0.95 (0.80–1.13)	0.54	0.96 (0.81–1.14)	0.66	0.89 (0.76–1.06)	0.19	0.94 (0.80–1.11)	0.46
Site of data collection								
Public Spaces	Ref	Ref	Ref	Ref	Ref	Ref	Ref	Ref
Hospitals	1.65 (1.42–1.93)	< 0.001	1.85 (1.58–2.15)	< 0.001	1.78 (1.54–2.07)	< 0.001	1.65 (1.43–1.90)	< 0.001
Primary healthcare centers	2.07 (1.72–2.48)	< 0.001	2.09 (1.75–2.49)	< 0.001	1.79 (1.50–2.12)	< 0.001	1.70 (1.44–2.01)	< 0.001

AOR = adjusted odds ratio, CI = confidence interval, WBJ = West Bank and Jerusalem

*Adjusted for age-group, gender, educational level, occupation, monthly income, marital status, residency, having a chronic disease, knowing someone with cancer, smoking history, and site of data collection

### Association between recognizing non-respiratory LC symptoms and participant characteristics

Participants recruited from primary healthcare centers were more likely than participants recruited from public spaces to recognize all non-respiratory LC symptoms (Table 6). In addition, participants recruited from hospitals were more likely than participants recruited from public spaces to recognize all non-respiratory LC symptoms except ‘changes in the shape of fingers or nails’ and ‘persistent shoulder pain’ for which no associated differences were noticed. Participants who knew someone with cancer were more likely than participants who did not to recognize all non-respiratory LC symptoms except ‘developing an unexplained loud, high-pitched sound when breathing’ for which no associated difference was found. Female participants and those who completed post-secondary education were more likely to recognize three of the six non-respiratory LC symptoms. There were no associated differences in the likelihood to recognize all non-respiratory LC symptoms between smokers and non-smokers.

**Table 6:** Multivariable logistic regression analyzing factors associated with the recognition of non-respiratory symptoms of lung cancer

Characteristic	Persistent tiredness or lack of energy		Developing an unexplained loud, high-pitched sound when breathing		Loss of appetite		Unexplained weight loss		Changes in the shape of fingers or nails		Persistent shoulder pain	
	AOR (95% CI)*	p-value	AOR (95% CI)*	p-value	AOR (95% CI)*	p-value	AOR (95% CI)*	p-value	AOR (95% CI)*	p-value	AOR (95% CI)*	p-value
Age group												
18 to 44	Ref	Ref	Ref	Ref	Ref	Ref	Ref	Ref	Ref	Ref	Ref	Ref
45 or older	0.98 (0.82–1.16)	0.77	0.98 (0.83–1.17)	0.86	0.96 (0.81–1.13)	0.60	1.28 (1.08–1.51)	0.004	1.07 (0.90–1.27)	0.43	1.22 (1.01–1.47)	0.036
Gender												
Male	Ref	Ref	Ref	Ref	Ref	Ref	Ref	Ref	Ref	Ref	Ref	Ref
Female	1.06 (0.89–1.27)	0.50	1.23 (1.04–1.47)	0.018	1.09 (0.92–1.29)	0.33	1.30 (1.10–1.54)	0.002	1.11 (0.94–1.32)	0.23	1.43 (1.18–1.74)	< 0.001
Educational level												
Secondary or below	Ref	Ref	Ref	Ref	Ref	Ref	Ref	Ref	Ref	Ref	Ref	Ref
Above secondary	1.41 (1.23–1.62)	< 0.001	1.31 (1.15–1.50)	< 0.001	1.12 (0.98–1.27)	0.12	1.23 (1.08–1.40)	0.002	0.88 (0.77–1.01)	0.07	1.08 (0.93–1.26)	0.33
Occupation												
Unemployed/housewife	Ref	Ref	Ref	Ref	Ref	Ref	Ref	Ref	Ref	Ref	Ref	Ref
Employed	1.13 (0.96–1.34)	0.15	1.20 (1.02–1.42)	0.031	0.92 (0.78–1.08)	0.32	1.24 (1.06–1.46)	0.007	0.97 (0.82–1.14)	0.70	1.07 (0.89–1.28)	0.48
Retired	1.26 (0.80–1.98)	0.32	0.99 (0.65–1.50)	0.94	1.08 (0.70–1.66)	0.75	1.09 (0.72–1.67)	0.68	0.83 (0.53–1.28)	0.40	0.85 (0.52–1.39)	0.51
Student	1.31 (1.01–1.69)	0.043	1.20 (0.93–1.54)	0.16	1.01 (0.79–1.29)	0.92	1.09 (0.86–1.38)	0.48	1.18 (0.92–1.50)	0.20	1.04 (0.79–1.37)	0.77
Monthly income												
< 1450 NIS	Ref	Ref	Ref	Ref	Ref	Ref	Ref	Ref	Ref	Ref	Ref	Ref
≥ 1450 NIS	1.07 (0.89–1.28)	0.46	1.03 (0.86–1.22)	0.77	1.00 (0.84–1.19)	0.97	1.09 (0.92–1.29)	0.31	0.84 (0.71–1.00)	0.048	1.25 (1.04–1.52)	0.021
Marital status												
Single	Ref	Ref	Ref	Ref	Ref	Ref	Ref	Ref	Ref	Ref	Ref	Ref
Married	1.01 (0.86–1.19)	0.91	0.89 (0.76–1.04)	0.13	1.18 (1.01–1.38)	0.034	1.15 (.99–1.34)	0.06	1.02 (0.87–1.19)	0.85	0.87 (0.73–1.04)	0.13
Divorced/Widowed	0.87 (0.61–1.26)	0.47	1.15 (0.79–1.67)	0.48	1.17 (.81–1.68)	0.40	1.03 (0.72–1.46)	0.89	0.89 (0.62–1.29)	0.54	1.00 (.68–1.48)	1.00
Residency												
Gaza Strip	Ref	Ref	Ref	Ref	Ref	Ref	Ref	Ref	Ref	Ref	Ref	Ref
WBJ	0.83 (0.70–0.98)	0.032	1.12 (0.95–1.32)	0.17	0.91 (0.78–1.07)	0.27	1.02 (0.87–1.19)	0.81	0.86 (0.73–1.01)	0.06	0.71 (0.59–0.85)	< 0.001
Having a chronic disease												
No	Ref	Ref	Ref	Ref	Ref	Ref	Ref	Ref	Ref	Ref	Ref	Ref
Yes	0.98 (0.82–1.16)	0.81	1.00 (0.84–1.18)	0.98	0.92 (0.781.09)	0.35	0.98 (0.83–1.15)	0.79	0.99 (0.83–1.17)	0.88	1.23 (1.03–1.48)	0.026
Knowing someone with cancer												
No	Ref	Ref	Ref	Ref	Ref	Ref	Ref	Ref	Ref	Ref	Ref	Ref
Yes	1.55 (1.37–1.76)	< 0.001	1.06 (0.94–1.20)	0.35	1.59 (1.40–1.79)	< 0.001	1.44 (1.28–1.63)	< 0.001	1.42 (1.26–1.61)	< 0.001	1.15 (1.00–1.32)	0.043
Ever smoked cigarettes and/or shisha												
No	Ref	Ref	Ref	Ref	Ref	Ref	Ref	Ref	Ref	Ref	Ref	Ref
Yes	0.92 (0.78–1.09)	0.32	0.91 (0.77–1.07)	0.24	1.01 (0.86–1.18)	0.95	1.02 (0.87–1.19)	0.80	0.89 (0.75–1.04)	0.15	0.88 (0.72–1.06)	0.17
Site of data collection												
Public Spaces	Ref	Ref	Ref	Ref	Ref	Ref	Ref	Ref	Ref	Ref	Ref	Ref
Hospitals	1.64 (1.42–1.90)	< 0.001	1.93 (1.67–2.23)	< 0.001	1.22 (1.06–1.41)	0.005	1.49 (1.30–1.72)	< 0.001	1.08 (0.94–1.25)	0.23	1.03 (0.87–1.22)	0.71
Primary healthcare	1.93 (1.62–2.29)	< 0.001	1.98 (1.68–2.35)	< 0.001	1.56 (1.32–1.84)	< 0.001	1.74 (1.48–2.05)	< 0.001	1.08 (0.92–1.28)	0.34	1.85 (1.55–2.21)	< 0.001

AOR = adjusted odds ratio, CI = confidence interval, WBJ = West Bank and Jerusalem

*Adjusted for age-group, gender, educational level, occupation, monthly income, marital status, residency, having a chronic disease, knowing someone with cancer, smoking history, and site of data collection

## Discussion

More than half of the study participants (51.8%) displayed good awareness of LC symptoms. Participants from both the Gaza Strip and the WBJ had a similar likelihood to have good awareness of LC symptoms. Factors associated with displaying good awareness included female gender, having post-secondary education, being employed, knowing someone with cancer, and visiting hospitals and primary healthcare centers. The most recognized respiratory LC symptom was ‘worsening or change in an existing cough’, whereas the most recognized non-respiratory LC symptom was ‘persistent tiredness or lack of energy'. The least recognized respiratory LC symptom was ‘a cough that does not go away for two or three weeks’, whereas the least recognized non-respiratory LC symptom was ‘persistent shoulder pain’.

Good awareness of LC symptoms was shown to be associated with a shorter time to seek medical advice, which can facilitate diagnosis of LC at early stages [15–17, 35]. Therefore, the determination of the public awareness of LC symptoms is crucial to identify the knowledge gap that needs to be addressed by educational interventions. This study assessed LC symptoms awareness among Palestinians to provide a baseline that can help in designing future educational interventions. Such interventions are of special importance in low-resource settings such as Palestine; given the shortage of diagnosis and treatment modalities [38].

### Awareness of LC symptoms

The results of this study indicate that there is room for improving the public awareness of LC symptoms in Palestine. Suboptimal levels of knowledge of LC symptoms were also found in Australia, Nigeria and the United Kingdom [11, 36, 37]. This may highlight the crucial need for standardized educational interventions to raise public awareness of LC symptoms by world health authorities particularly as LC is a major contributor to the global burden of cancer-related morbidity and mortality [2].

The proportion of Palestinians who displayed good levels of awareness of LC symptoms (51.8%) was higher than the proportion who was aware of cervical (27.4%) and ovarian cancer symptoms (17.4%) [22–24]. This might reflect greater familiarity with respiratory symptoms than with those related to gynecological cancers. The public might feel less embarrassed to read or discuss respiratory symptoms they might experience compared to reading or discussing symptoms related to gynecological cancers. In addition, topics related to gynecological health would be of greater interest to women only, while LC would be a subject of interest to both men and women. A further contributing factor to the higher awareness of LC symptoms could be the high prevalence of smoking in Palestine especially among men [18]. It is possible that the public is well aware of smoking as a risk factor for LC, therefore, there could be more interest to read more about LC than other cancers. Interestingly, women in this study were more likely than men to have good knowledge of LC symptoms. Women, especially these who are non-smokers, are more susceptible to develop LC [39]. Palestinian women might be aware of this, which could drive them to improve their health literacy about LC. Further studies are needed to assess the recognition of LC risk factors and to examine the differences in awareness between smokers and non-smokers as well as between men and women.

In this study, ‘a cough that does not go away for two or three weeks’ was the least recognized respiratory symptom of LC, which is similar to findings of another study conducted in the United Kingdom [11]. It could be that participants might have related the presence of persistent cough to reasons other than LC, such as benign lung diseases (e.g., chronic obstructive pulmonary disease) or smoking [40, 41]. Conversely, ‘coughing up blood’ was one of the most recognized respiratory symptoms similar to findings of other studies from Australia, Nigeria and Canada [36, 37, 42]. Previous studies from Palestine showed that participants were more able to recognize cancer symptoms if they were associated with bleeding [22, 25, 26]. Forbes and colleagues found that patients with bleeding symptoms had a higher likelihood of seeking medical advice earlier than patients who did not complain of bleeding symptoms [43].

Similar to a study conducted in the United Kingdom [11], the two least recognized non-respiratory symptoms of LC in this study were ‘persistent shoulder pain’ and ‘changes in the shape of fingers or nails’. Future educational interventions should focus more on the differing nature of LC symptoms with the emphasis that these symptoms can be non-respiratory [44].

### Factors associated with good awareness of LC symptoms

In line with other studies from Nigeria and the United Kingdom [11, 36], the completion of post-secondary education was associated with an increase in the likelihood of displaying good awareness of LC in this study. More educated participants might have a higher possibility for meeting or working with people who have a good background of health-related topics. Similarly, knowing someone with cancer was associated with a higher likelihood of having good awareness of LC symptoms, which comes in concordance with other studies [12, 25, 41]. Social interactions in Palestine seem to play a role in accumulating more knowledge about health-related topic [22–24]. Female participants had a higher likelihood to display good awareness of LC symptoms, which was also found in a study from Australia [37]. In Palestine, women tend to have more visits to healthcare facilities than men; mainly because they access maternal and child healthcare services. This might have helped women to shape better health literacy. In addition, women more often take care of their sick relatives. Therefore, it is possible that women might get more exposed to different experiences of relatives diagnosed with cancer [22–24].

Previous studies assessing awareness of ovarian and cervical cancers in Palestine found differences in the awareness levels between the Gaza Strip and the WBJ [22, 24]. In this study, the overall awareness between participants from the Gaza Strip and WBJ was similar. This suggests the potential equity in the benefits from applying standardized educational interventions that aim to raise the public awareness about LC symptoms across Palestine. Previous studies demonstrated the positive impact of raising the public awareness of cancer symptoms on seeking medical advice and improving early diagnosis of LC [16, 17]. This is especially important in low- and middle-income countries, such as Palestine, where cancer deaths are on a steady increase and predicted to rise to make up 75% of global cancer deaths by 2030, while cancer mortality in high-income countries is either stable or declining [3]. This decline has largely been achieved by applied prevention strategies, such as anti-smoking campaigns, earlier detection and improved treatment [3]. Often, access to and availability of effective treatment modalities are scarce in low- and middle-income countries, therefore, early detection of cancer is the linchpin to improved cancer control in these countries [3]. Good awareness of cancer symptoms and risk factors can contribute to early presentation and, thus, earlier diagnosis [3, 11]. LC is the leading cause of cancer-related deaths in Palestine as well as globally [1, 2, 5]. However, evidence of LC awareness in low- and middle-income countries or interventions to improve awareness are scarce [45]. This research contributes to the body of evidence on LC awareness in Palestine as well as in the region. The health authorities and policy makers in Palestine can use these data to tailor their efforts in the context of the Palestinian community to provide a holistic approach to promote people’s health literacy [15–17, 46]. For example, this study found that smokers and non-smokers had a similar likelihood to recognize respiratory and non-respiratory symptoms of LC. However, smokers are at increased risk to develop LC [47, 48]. Therefore, it is important that health policy makers in Palestine focus on the recognition of LC risk factors while establishing educational interventions and effective tobacco control policies to increase risk awareness, change behaviors and encourage early recognition and presentation. With targeted awareness campaigns, smokers might realize their increased risk to develop LC and seek medical advice earlier for any possible LC symptoms they might experience. In addition, these campaigns may help correct one of the common misbeliefs among smokers that respiratory symptoms are only attributed to smoking itself neglecting the likelihood of having LC as a possible diagnosis [37, 49]. Addressing smokers’ fear, self-blame and denial of smoking-related diseases, including LC, should also be considered while establishing these campaigns [37, 50–52].

More research is needed in Palestine as well as the wider region on the sources of information used by the public to be able to distribute information effectively via popular channels for different population groups such as younger males or the older age groups. Furthermore, barriers to health-seeking behavior need to be explored in order to address them and facilitate earlier presentation to healthcare facilities [15, 17, 25]. This might be achieved by exploring experiences and behaviors of patients who have been diagnosed with LC [53]. Moreover, more investment in cancer prevention and control in low- and middle-income countries, such as Palestine, is crucial but often avoided due to its costs [3]. However, campaigns focused on LC awareness and prevention, such as anti-smoking messages and tobacco control policies are potentially low-cost and high-impact interventions that could have positive impact on risk factor modification and early presentation.

### Strengths and limitations

The major strengths of this study include the high response rate, the large sample size, and the enrolment of participants from different places across Palestine. In addition, the completion of the questionnaire was done in a face-to-face interview, which minimizes the possibility of using external sources (e.g., the internet) to answer questions.

This study has some limitations. The use of convenience sampling does not guarantee the generation of a representative sample and limits the generalizability of the results. However, this might have been mitigated by the large number of participants included, the high response rate, and the recruitment from various geographical places. For example, females made up 55% of the participants in this study while they represent about 50% of the Palestinian population [54]. In addition, the lower monthly income among participants from the Gaza Strip than that of participants from the WBJ reflected the difference in the unemployment rates that are higher in the Gaza Strip than in the WBJ (47% vs. 16%) [55]. Moreover, the demographics of the participants of this study were very close to the demographics reported by other studies conducted in Palestine to assess knowledge about various topics related to cancer [22–24]. Another limitation could be that most of the study participants were young (< 45 years) and so, they had a relatively lower risk of developing LC. This higher proportion of younger participants could be due to the fact that they represent the majority of the Palestinian population [54]. Nevertheless, improving the awareness among young individuals might be an effective strategy to build up a culture of early recognition of LC symptoms and seeking prompt medical advice for any possible LC symptoms. Finally, the exclusion of participants with medical background and with a presumably good awareness of LC symptoms might have lowered the awareness observed in this study. Nonetheless, their exclusion was intended to make this study more relevant as a measure of public awareness.

## Conclusions

Around half of the participants (51.8%) displayed good awareness of LC symptoms in Palestine. Participants from both the Gaza Strip and the WBJ were similarly likely to display good awareness. Factors associated with good awareness included female gender, having benefited from post-secondary education, being employed, knowing someone with cancer, and visiting hospitals and primary healthcare centers. The most recognized respiratory LC symptom was ‘worsening or change in an existing cough’, whereas ‘persistent tiredness or lack of energy' was the most recognized non-respiratory LC symptom. This study highlights the need for awareness and education programs and campaigns, especially in low- and middle-income countries like Palestine, to improve awareness and thus reduce the chances of late detection of LC.

## Supplementary Information

**Additional file 1**. Questionnaire.

**Additional file 2**. Results of bivariable analyses.

## Data Availability

The dataset used and analyzed during the current study is available from the corresponding author on reasonable request.

## Abbreviations

LC: Lung cancer
LCAM: Lung cancer awareness measure
WHO: World Health Organization
WBJ: West Bank and Jerusalem
MoH: Ministry of health
IQR: Interquartile range
CI: Confidence interval
OR: Odds ratio
