# Supplementary material for: Awareness of Palestinians about lung cancer symptoms: a national cross-sectional study
Source: BMC Pulm Med. 2022 Apr 8;22:135. doi: 10.1186/s12890-022-01923-1 (PMC8991725; doi:10.1186/s12890-022-01923-1)
Supplement: Supplementary file 1 — Additional file 1. Questionnaire. [file 12890_2022_1923_MOESM1_ESM.docx]

**Lung Cancer Awareness Questionnaire**

Serial number: ………. Location: …………. Governorate: ……….

- Do you know someone diagnosed with cancer? 🞏 No 🞏 Yes
- Have you ever smoked cigarettes?

🞏 Never smoked 🞏 Former smoker 🞏 Current smoker

- Have you ever smoked waterpipe tobacco?

🞏 Never smoked 🞏 Former smoker 🞏 Current smoker

| Table (1): The following may or may not be warning signs for lung cancer. We are interested in your opinion: | | | | | |
| --- | --- | --- | --- | --- | --- |
| Symptom/sign | **1= Strongly Disagree** | **2= Disagree** | **3=**  **Not Sure** | **4= Agree** | **5= Strongly agree** |
| 1. Unexplained weight loss |  |  |  |  |  |
| 1. Persistent (3 weeks or longer) chest infection |  |  |  |  |  |
| 1. A cough that does not go away for two or three weeks |  |  |  |  |  |
| 1. Persistent shortness of breath |  |  |  |  |  |
| 1. Persistent tiredness or lack of energy |  |  |  |  |  |
| 1. Persistent chest pain |  |  |  |  |  |
| 1. Persistent shoulder pain |  |  |  |  |  |
| 1. Coughing up blood |  |  |  |  |  |
| 1. An ache or pain when breathing |  |  |  |  |  |
| 1. Loss of appetite |  |  |  |  |  |
| 1. Painful cough |  |  |  |  |  |
| 1. Changes in the shape of your fingers or nails |  |  |  |  |  |
| 1. Developing an unexplained loud, high-pitched sound when breathing |  |  |  |  |  |
| 1. Worsening or change in an existing cough |  |  |  |  |  |

**Sociodemographic Data**

- Age: …….. years
- Gender: 🞏 Male 🞏 Female
- Marital status: 🞏 Single 🞏 Married 🞏 Divorced 🞏 Widowed
- Highest level of education: 🞏 Secondary or below 🞏 Post-secondary
- Occupation: 🞏 Unemployed/Housewife 🞏 Employee 🞏 Retired 🞏 Student
- Monthly income (NIS): ………………
- Do you have any chronic disease? 🞏 No 🞏 Yes
