# Supplementary material for: Awareness of Palestinians about lung cancer symptoms: a national cross-sectional study
Source: BMC Pulm Med. 2022 Apr 8;22:135. doi: 10.1186/s12890-022-01923-1 (PMC8991725; doi:10.1186/s12890-022-01923-1)
Supplement: Supplementary file 2 — Additional file 2. Results of bivariable analyses. [file 12890_2022_1923_MOESM2_ESM.docx]

| Characteristic  Supplementary table 1: Bivariable logistic regression analyzing factors associated with the recognition of respiratory symptoms of lung cancer. | Worsening or change in an existing cough | | Coughing up blood | | Persistent shortness of breath | | An ache or pain when breathing | |
| --- | --- | --- | --- | --- | --- | --- | --- | --- |
|  | **COR (95% CI)** | **p-value** | **COR (95% CI)** | **p-value** | **COR (95% CI)** | **p-value** | **COR (95% CI)** | **p-value** |
| Age group  18 to 44  45 or older | Ref  0.76 (0.65-0.90) | Ref  0.001 | Ref  0.91 (0.77-1.06) | Ref  0.23 | Ref  0.86 (0.74-1.00) | Ref  0.042 | Ref  0.74 (0.64-0.86) | Ref  <0.001 |
| Gender  Male Female | Ref  1.31 (1.14-1.52) | Ref  <0.001 | Ref  1.06 (0.93-1.23) | Ref  0.34 | Ref  0.96 (0.84-1.09) | Ref  0.52 | Ref  1.12 (0.99-1.28) | Ref  0.08 |
| Educational level  Secondary or below  Above secondary | Ref  1.84 (1.58-2.14) | Ref  <0.001 | Ref  1.53 (1.33-1.76) | Ref  <0.001 | Ref  1.77 (1.56-2.02) | Ref  <0.001 | Ref  1.49 (1.31-1.70) | Ref  <0.001 |
| Occupation  Unemployed/housewife  Employed  Retired  Student | Ref  1.01 (0.87-1.19)  0.63 (0.41-0.97)  1.52 (1.14-2.02) | Ref  0.86  0.037  0.004 | Ref  1.25 (1.08-1.46)  1.08 (0.68-1.72)  1.37 (1.07-1.77) | Ref  0.003  0.74  0.014 | Ref  1.17 (1.02-1.35)  1.37 (0.87-2.16)  1.71 (1.34-2.19) | Ref  0.023  0.18  <0.001 | Ref  1.06 (0.93-1.22)  1.16 (0.75-1.80)  1.46 (1.15-1.86) | Ref  0.38  0.51  0.002 |
| Monthly income  < 1450 NIS  ≥ 1450 NIS | Ref  1.34 (1.15-1.56) | Ref  <0.001 | Ref  1.46 (1.27-1.69) | Ref  <0.001 | Ref  1.27 (1.11-1.45) | Ref  0.001 | Ref  1.12 (0.98-1.28) | Ref  0.11 |
| Marital status  Single  Married  Divorced/Widowed | Ref  0.80 (0.68-0.94)  0.74 (0.50-1.11) | Ref  0.008  0.15 | Ref  0.98 (0.84-1.14)  1.33 (0.86-2.04) | Ref  0.77  0.20 | Ref  0.90 (0.78-1.03)  0.61 (0.43-0.85) | Ref  0.13  0.004 | Ref  0.79 (0.69-0.91)  0.67 (0.47-0.95) | Ref  0.001  0.025 |
| Residency  Gaza Strip  WBJ | Ref  1.21 (1.04-1.40) | Ref  0.011 | Ref  1.41 (1.22-1.62) | Ref  <0.001 | Ref  1.06 (0.93-1.21) | Ref  0.37 | Ref  1.04 (0.91-1.19) | Ref  0.54 |
| Having a chronic disease  No  Yes | Ref  1.02 (0.85-1.22) | Ref  0.84 | Ref  1.02 (0.86-1.21) | Ref  0.82 | Ref  0.85 (0.73-0.99) | Ref  0.033 | Ref  0.77 (0.66-0.90) | Ref  0.001 |
| Knowing someone with cancer  No  Yes | Ref  1.18 (1.02-1.36) | Ref  0.030 | Ref  1.03 (0.90-1.19) | Ref  0.65 | Ref  1.26 (1.11-1.44) | Ref  <0.001 | Ref  1.26 (1.11-1.44) | Ref  <0.001 |
| Ever smoked cigarettes and/or shisha  No  Yes | Ref  0.81 (0.69-0.95) | Ref  0.008 | Ref  0.98 (0.84-1.14) | Ref  0.80 | Ref  0.95 (0.82-1.09) | Ref  0.43 | Ref  0.91 (0.79-1.04) | Ref  0.16 |
| Site of data collection  Public Spaces  Hospitals  Primary healthcare centers | Ref  1.21 (1.02-1.43)  1.43 (1.18-1.73) | Ref  0.029  <0.001 | Ref  1.22 (1.04-1.44)  1.30 (1.09-1.56) | Ref  0.015  0.004 | Ref  1.46 (1.26-1.70)  1.54 (1.30-1.82) | Ref  <0.001  <0.001 | Ref  1.47 (1.27-1.70)  1.68 (1.42-1.98) | Ref  <0.001  <0.001 |

COR= crude odds ratio, CI= confidence interval, WBJ= West Bank and Jerusalem.

Supplementary table 1: Bivariable logistic regression analyzing factors associated with the recognition of respiratory symptoms of lung cancer. (Ctd)

| Characteristic | Persistent chest pain | | Painful cough | | Persistent (3 weeks or longer) chest infection | | A cough that does not go away for two or three weeks | |
| --- | --- | --- | --- | --- | --- | --- | --- | --- |
|  | **COR (95% CI)** | **p-value** | **COR (95% CI)** | **p-value** | **COR (95% CI)** | **p-value** | **COR (95% CI)** | **p-value** |
| Age group  18 to 44  45 or older | Ref  1.01 (0.88-1.17) | Ref  0.86 | Ref  0.88 (0.76-1.01) | Ref  0.07 | Ref  0.91 (0.79-1.04) | Ref  0.17 | Ref  0.98 (0.85-1.12) | Ref  0.71 |
| Gender  Male Female | Ref  0.97 (0.85-1.10) | Ref  0.64 | Ref  1.12 (0.99-1.27) | Ref  0.09 | Ref  1.04 (0.92-1.18) | Ref  0.53 | Ref  1.09 (0.97-1.23) | Ref  0.14 |
| Educational level  Secondary or below  Above secondary | Ref  1.31 (1.15-1.48) | Ref  <0.001 | Ref  1.51 (1.33-1.71) | Ref  <0.001 | Ref  1.58 (1.40-1.79) | Ref  <0.001 | Ref  1.52 (1.36-1.72) | Ref  <0.001 |
| Occupation  Unemployed/housewife  Employed  Retired  Student | Ref  1.08 (0.94-1.23)  1.35 (0.86-2.12)  1.39 (1.10-1.75) | Ref  0.29  0.19  0.006 | Ref  1.05 (0.92-1.20)  0.96 (0.64-1.46)  1.47 (1.17-1.86) | Ref  0.47  0.86  0.001 | Ref  1.14 (1.00-1.30)  1.14 (0.75-1.72)  1.34 (1.07-1.67) | Ref  0.053  0.55  0.009 | Ref  1.18 (1.04-1.33)  1.13 (0.76-1.67)  1.43 (1.16-1.77) | Ref  0.011  0.55  0.001 |
| Monthly income  < 1450 NIS  ≥ 1450 NIS | Ref  1.10 (0.97-1.26) | Ref  0.15 | Ref  1.31 (1.15-1.49) | Ref  <0.001 | Ref  1.20 (1.05-1.37) | Ref  0.006 | Ref  1.33 (1.17-1.50) | Ref  <0.001 |
| Marital status  Single  Married  Divorced/Widowed | Ref  0.90 (0.78-1.03)  0.79 (0.56-1.13) | Ref  0.14  0.20 | Ref  0.84 (0.73-0.96)  0.74 (0.52-1.04) | Ref  0.010  0.08 | Ref  0.90 (0.78-1.03)  0.57 (0.41-0.79) | Ref  0.110  0.001 | Ref  0.87 (0.76-0.98)  0.79 (0.57-1.10) | Ref  0.026  0.16 |
| Residency  Gaza Strip  WBJ | Ref  0.97 (0.85-1.11) | Ref  0.66 | Ref  1.23 (1.09-1.40) | Ref  0.001 | Ref  1.05 (0.92-1.18) | Ref  0.49 | Ref  1.24 (1.10-1.39) | Ref  <0.001 |
| Having a chronic disease  No  Yes | Ref  0.96 (0.82-1.12) | Ref  0.61 | Ref  0.94 (0.81-1.09) | Ref  0.38 | Ref  0.87 (0.75-1.00) | Ref  0.053 | Ref  0.95 (0.83-1.10) | Ref  0.49 |
| Knowing someone with cancer  No  Yes | Ref  1.30 (1.14-1.47) | Ref  <0.001 | Ref  1.24 (1.10-1.41) | Ref  0.001 | Ref  1.12 (0.99-1.27) | Ref  0.07 | Ref  1.09 (0.97-1.23) | Ref  0.14 |
| Ever smoked cigarettes and/or shisha  No  Yes | Ref  0.98 (0.85-1.12) | Ref  0.75 | Ref  0.93 (0.81-1.06) | Ref  0.26 | Ref  0.90 (0.79-1.03) | Ref  0.12 | Ref  0.92 (0.81-1.05) | Ref  0.22 |
| Site of data collection  Public Spaces  Hospitals  Primary healthcare centers  COR= crude odds ratio, CI= confidence interval, WBJ= West Bank and Jerusalem. | Ref  1.47 (1.27-1.70)  1.63 (1.39-1.92) | Ref  <0.001  <0.001 | Ref  1.57 (1.36-1.82)  1.65 (1.41-1.94) | Ref  <0.001  <0.001 | Ref  1.57 (1.36-1.81)  1.48 (1.27-1.73) | Ref  <0.001  <0.001 | Ref  1.44 (1.26-1.65)  1.40 (1.21-1.63) | Ref  <0.001  <0.001 |

| Characteristic | Persistent tiredness or lack of energy  Supplementary table 2: Bivariable logistic regression analyzing factors associated with the recognition of non-respiratory symptoms of lung cancer. | | Developing an unexplained loud, high-pitched sound when breathing | | Loss of appetite | | Unexplained weight loss | | Changes in the shape of fingers or nails | | Persistent shoulder pain | |
| --- | --- | --- | --- | --- | --- | --- | --- | --- | --- | --- | --- | --- |
|  | **COR (95% CI)** | **p-value** | **COR (95% CI)** | **p-value** | **COR (95% CI)** | **p-value** | **COR (95% CI)** | **p-value** | **COR (95% CI)** | **p-value** | **COR (95% CI)** | **p-value** |
| Age group  18 to 44  45 or older | Ref  0.91 (0.80-1.05) | Ref  0.20 | Ref  0.93 (0.81-1.07) | Ref  0.31 | Ref  1.00 (0.87-1.15) | Ref  1.00 | Ref  1.32 (1.15-1.51) | Ref  <0.001 | Ref  1.05 (0.91-1.20) | Ref  0.53 | Ref  1.21 (1.04-1.40) | Ref  0.014 |
| Gender  Male Female | Ref  1.08 (0.96-1.22) | Ref  0.19 | Ref  1.25 (1.11-1.40) | Ref  <0.001 | Ref  1.21 (1.08-1.37) | Ref  0.001 | Ref  1.22 (1.08-1.36) | Ref  0.001 | Ref  1.21 (1.07-1.37) | Ref  0.002 | Ref  1.69 (1.47-1.94) | Ref  <0.001 |
| Educational level  Secondary or below  Above secondary | Ref  1.41 (1.25-1.59) | Ref  <0.001 | Ref  1.28 (1.14-1.44) | Ref  <0.001 | Ref  1.04 (0.92-1.17) | Ref  0.52 | Ref  1.13 (1.01-1.27) | Ref  0.035 | Ref  0.87 (0.77-0.98) | Ref  0.019 | Ref  1.04 (0.91-1.19) | Ref  0.57 |
| Occupation  Unemployed/housewife  Employed  Retired  Student | Ref  1.02 (0.89-1.16)  1.24 (0.81-1.88)  1.18 (0.96-1.47) | Ref  0.79  0.33  0.13 | Ref  0.99 (0.87-1.12)  0.83 (0.56-1.23)  1.10 (0.89-1.35) | Ref  0.85  0.35  0.38 | Ref  0.81 (0.71-0.92)  1.02 (0.68-1.54)  0.80 (0.65-0.98) | Ref  0.001  0.91  0.035 | Ref  1.02 (0.90-1.15)  1.15 (0.78-1.70)  0.80 (0.65-0.97) | Ref  0.75  0.48  0.024 | Ref  0.80 (0.70-0.90)  0.74 (0.49-1.12)  0.94 (0.77-1.16) | Ref  <0.001  0.15  0.57 | Ref  0.73 (0.63-0.84)  0.72 (0.45-1.14)  0.82 (0.65-1.03) | Ref  <0.001  0.16  0.09 |
| Monthly income  < 1450 NIS  ≥ 1450 NIS | Ref  0.96 (0.85-1.10) | Ref  0.58 | Ref  1.07 (0.94-1.21) | Ref  0.33 | Ref  0.91 (0.80-1.04) | Ref  0.15 | Ref  1.14 (1.01-1.29) | Ref  0.036 | Ref  0.74 (0.65-0.84) | Ref  <0.001 | Ref  0.92 (0.80-1.06) | Ref  0.27 |
| Marital status  Single  Married  Divorced/Widowed | Ref  1.02 (0.89-1.16)  0.77 (0.55-1.07) | Ref  0.77  0.12 | Ref  0.96 (0.85-1.09)  1.14 (0.81-1.61) | Ref  0.55  0.45 | Ref  1.24 (1.09-1.41)  1.17 (0.84-1.63) | Ref  0.001  0.37 | Ref  1.36 (1.20-1.54)  1.25 (0.90-1.73) | Ref  <0.001  0.18 | Ref  1.06 (0.93-1.20)  1.03 (0.73-1.44) | Ref  0.42  0.89 | Ref  1.09 (0.94-1.26)  1.39 (0.97-1.98) | Ref  0.24  0.07 |
| Residency  Gaza Strip  WBJ | Ref  0.85 (0.75-0.97) | Ref  0.011 | Ref  1.11 (0.99-1.25) | Ref  0.08 | Ref  0.90 (0.80-1.01) | Ref  0.07 | Ref  1.11 (0.99-1.25) | Ref  0.08 | Ref  0.78 (0.69-0.88) | Ref  <0.001 | Ref  0.82 (0.72-0.94) | Ref  0.004 |
| Having a chronic disease  No  Yes | Ref  0.93 (0.81-1.08) | Ref  0.35 | Ref  0.96 (0.83-1.11) | Ref  0.57 | Ref  1.00 (0.86-1.15) | Ref  0.96 | Ref  1.17 (1.02-1.34) | Ref  0.030 | Ref  1.04 (0.90-1.20) | Ref  0.61 | Ref  1.29 (1.11-1.51) | Ref  0.001 |
| Knowing someone with cancer  No  Yes | Ref  1.40 (1.24-1.58) | Ref  <0.001 | Ref  0.97 (0.86-1.09) | Ref  0.62 | Ref  1.49 (1.32-1.68) | Ref  <0.001 | Ref  1.38 (1.23-1.55) | Ref  <0.001 | Ref  1.36 (1.20-1.53) | Ref  <0.001 | Ref  1.07 (0.94-1.23) | Ref  0.30 |
| Ever smoked cigarettes and/or shisha  No  Yes | Ref  0.89 (0.78-1.01) | Ref  0.07 | Ref  0.82 (0.72-0.93) | Ref  0.003 | Ref  0.90 (0.79-1.02) | Ref  0.10 | Ref  0.94 (0.83-1.06) | Ref  0.32 | Ref  0.81 (0.71-0.92) | Ref  0.001 | Ref  0.65 (0.56-0.76) | Ref  <0.001 |
| Site of data collection  Public Spaces  Hospitals  Primary healthcare centers | Ref  1.46 (1.27-1.69)  1.64 (1.40-1.92) | Ref  <0.001  <0.001 | Ref  1.74 (1.52-2.00)  1.82 (1.57-2.12) | Ref  <0.001  <0.001 | Ref  1.19 (1.04-1.36)  1.55 (1.33-1.80) | Ref  0.014  <0.001 | Ref  1.43 (1.25-1.63)  1.64 (1.41-1.90) | Ref  <0.001  <0.001 | Ref  1.06 (0.92-1.21)  1.14 (0.98-1.33) | Ref  0.44  0.08 | Ref  0.95 (0.81-1.12)  1.92 (1.63-2.25) | Ref  0.53  <0.001 |

COR= crude odds ratio, CI= confidence interval, WBJ= West Bank and Jerusalem.
